# Meta-analysis of the effectiveness of early endoscopic treatment of Acute biliary pancreatitis based on lightweight deep learning model

**DOI:** 10.1186/s12876-024-03361-1

**Published:** 2024-08-28

**Authors:** Rihui Xiong, Danjuan Xiong, Zhaoping Wu, Xifeng Xiao

**Affiliations:** 1 Department of Hepatopancreatobiliary Surgery, Jiujiang City Key Laboratory of Cell Therapy, Jiujiang NO.1 People’s Hospital, Jiujiang, 332000 Jiangxi China; 2 Department of Gastroenterology, Jiujiang City Key Laboratory of Cell Therapy, Jiujiang NO.1 People’s Hospital, Jiujiang, 332000 Jiangxi China

**Keywords:** Acute biliary pancreatitis, Early endoscopic retrograde cholangiopancreatography, Light-weight deep learning, Meta-analysis

## Abstract

**Background:**

Acute biliary pancreatitis (ABP) is a clinical common acute abdomen. After the first pancreatitis, relapse rate is high, which seriously affects human life and health and causes great economic burdens to family and society. According to a great many research findings, endoscopic retrograde cholangiopancreatography (ERCP) is an effective treatment method. However, whether ERCP should be performed in early stage of ABP is still controversial in clinical practice.

**Methods:**

Related articles were retrieved from Pubmed, Web of Science core library, Nature, Science Direct, and other databases published from January 2000 until now. The keywords included early ERCP, delayed ERCP, ABP, laparoscopy, and cholecystectomy, all which were connected by “or” and “and”. The language of articles was not restricted during the retrieval and Review Manager5.3 was employed to perform meta-analysis of experimental data. Finally, a total of 8 eligible articles were selected, including 8,801 patients.

**Results:**

The results of the meta-analysis demonstrated that no remarkable differences were detected in the incidence of complications, mortality, and operation time between patients undergoing ERCP in early stage and those receiving delayed ERCP. However, the hospitalization time of patients in experimental group was notably shorter than that among patients in control group.

**Conclusins:**

Early ERCP treatment is as safe as late ERCP treatment for biliary pancreatitis, and can significantly shorten the hospital stay. Hence, the therapy was worthy of clinical promotion. The research findings provided reference and basis for clinical treatment of relevant diseases.

**Supplementary Information:**

The online version contains supplementary material available at 10.1186/s12876-024-03361-1.

## Introduction

Acute pancreatitis (AP) is one of common clinical acute abdomens. According to epidemiological data, the global incidence of AP amounts to 4.9 ~ 73.4/100,000. There are various causes and 35–60% of AP results from bile duct diseases [1]. However, the main cause of AP is mild pancreatitis. 10–20% AP patients suffer from acute severe pancreatitis (ASP) combined with systemic inflammatory response syndrome, multiple organ dysfunction, and local or systemic complications. In most cases, AP is severe and the fatality is higher than 20%. Acute biliary pancreatitis (ABP) is defined as a general term for AP mainly caused by bile duct diseases. The main causes of ABP is bile duct obstruction caused by bile duct stones, ascaris lumbricoides, and hemorrhage [2]. In general, relieving biliopancreatic duct obstruction is the main principle and strategy of treating ABP and controlling further development of diseases. Therefore, early obstruction removal, drainage decompression, and regurgitation termination could improve ABP condition and reduce the incidence and fatality of complications [3].

In 1978, the treatment of ABP with endoscopic retrograde cholangiopancreatography (ERCP) combined with duodenal papillotomy was firstly reported. Some research demonstrated that ERCP itself resulted in postoperative complication with the incidence between 5% and 10% and the fatality between 0.02% and 0.5%. The commonest complication was pancreatitis after ERCP. According to some prospective randomized controlled trials (RCTs), ERCP could remarkably improve the prognosis for ABP in early stage of ABP versus conservative therapy and its safety and effectiveness had been verified. Later on, multiple clinical research into the treatment of ABP with early ERCP combined with enriched supportive therapy (EST) were reported successively. However, no consensus has been reached on the specific therapeutic effect of the above therapy [4]. A related meta-analysis revealed that ERCP could reduce the incidence of complications among patients with predicted severe ABP in early stage no matter they suffered from acute cholangitis and intestinal obstruction. However, mortality couldn’t be remarkably reduced [5]. In contrast, early ERCP had insignificant therapeutic effect on patients with mild ABP. Besides, ERCP combined with EST showed no effects on the incidence of complications and mortality of ABP patients. Recently, multiple clinical guidelines based on evidence-based analysis and RCT demonstrated that early ERCP was unsuitable for the patients with mean arterial blood pressure (MABP) combined with cholangitis or persistent bile duct obstruction. However, ERCP should be performed on patients within 24 h to 48 h after onset if they suffered from the above complications. Unfortunately, there were no specific treatment strategies for related treatment methods. In general, no unified conclusion has been drawn on the therapeutic effect of ERCP on ABP [6].

ERCP has high values in the diagnosis and treatment of pancreatitis. It is not only a treatment method but also a diagnosis approach. In recent years, it has been widely applied and developed in medical field with the continuous and rapid development of computer technology. Light-weight deep learning model is one of the recent noteworthy computer network technologies. According to the pathological images to be diagnosed, the model selected the samples similar to the image features from numerous databases. The selected samples came with their own labels to help doctors diagnose diseases. As a result, diagnostic efficiency and accuracy were dramatically improved. At present, the technology has been applied in related fields, such as the segmentation of tumor image lesions. In contrast, it has been rarely applied in the diagnosis and treatment of pancreatitis [7, 8]. Therefore, light-weight deep learning model was innovatively combined with ERCP and adopted for the diagnosis and treatment of pancreatitis. What’s more, the articles on the treatment of biliary pancreatitis with early ERCP were retrieved and collected. A meta-analysis of the safety of the above therapy was performed in terms of fatality, the incidence of complications, hospitalization duration, and operation time to provide reference and basis for clinical diagnosis and treatment of related diseases.

## Materials and methods

### Light-weight deep learning model

**Fig. 1:**
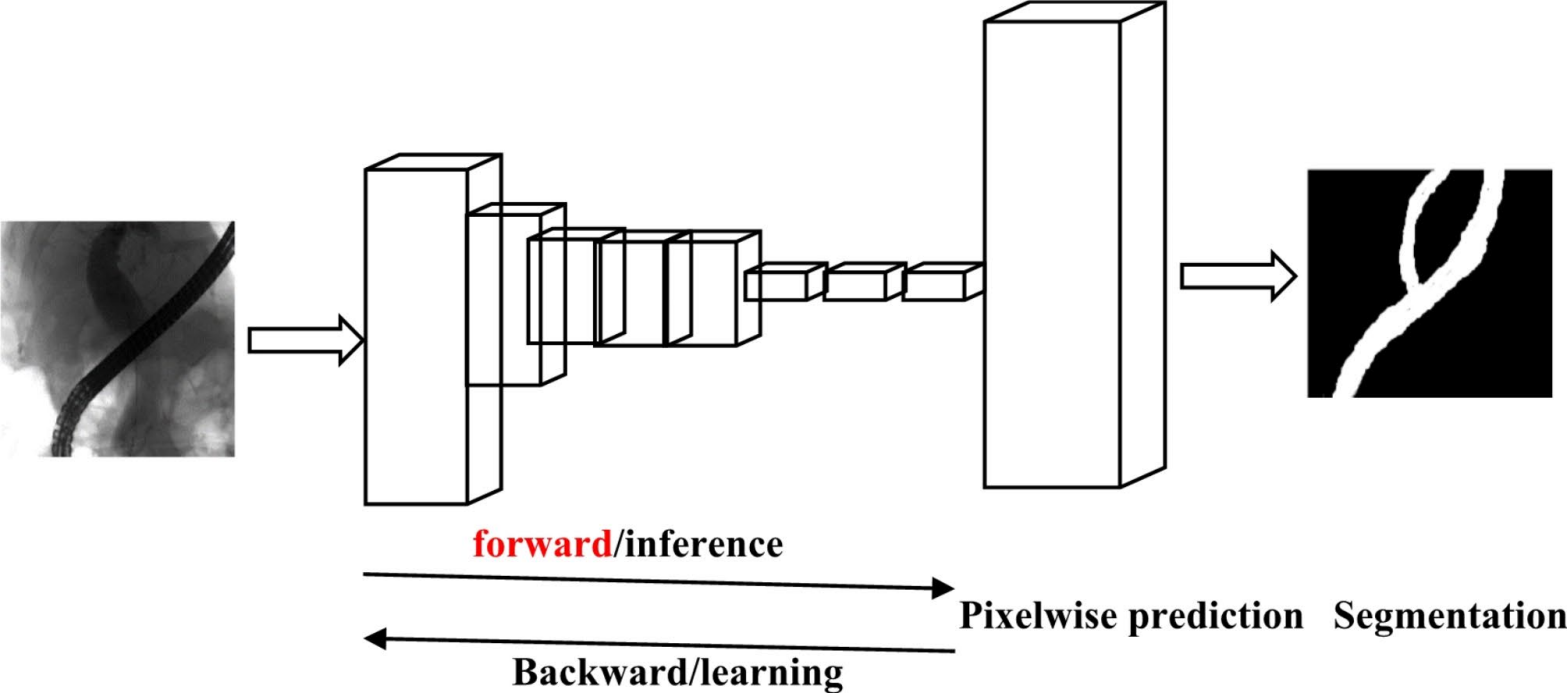
Structure of light-weight deep learning model

In terms of more efficient 3D extra light-weight ghost spatial pyramid net (GSPNet), expensive standard convolution was replaced with ghost convolution to reduce the computation amount and graphics processing unit (GPU) memory consumption of the network during feature extraction. Next, a light-weight GSP module was proposed. This module improved the representation power and multi-scale feature processing ability of the model by aggregating the features under different receptive fields. What’s more, pseudo-ghost bottleneck could be used to replace the standard convolution with maximum pooling or a step size of 2 to achieve down-sampling and avoid the loss of detail features and the increase of computation amount. GSPNet mainly consisted of ghost module, pseudo-ghost bottleneck, residual ghost module, and light-weight GSP module. The above modules were all light-weight feature processing modules. The complete network architecture was displayed in Fig. 1 below. It was demonstrated that GSPNet firstly used a layer of ghost module to augment channels for the input features. As a result, 4 channels were augmented to 32 channels in encoding stage. Secondly, pseudo-ghost bottleneck layer was utilized to replace the convolution with pooling or a step size of 2 to achieve down-sampling. After that, the proposed GSP module was employed to learn and fuse the features from different receptive fields. After the processing with GSP module, the number of channels increased again. In contracting path, GSPNet performed only 3 down-sampling and the maximum number of channels was kept at 256. In decoding stage, channel reduction and resolution recovery were carried out using 1 × 1 convolution for the features after up-sampling. Then, low-level detail features in the corresponding encoders were fused with high-level semantic features in the decoders by adding eigenvalues in the corresponding channel dimensions one by one. Next, the fused semantic features were refined by ghost modules including residual connection again to avoid network degradation. Finally, softmax function was utilized to convert each voxel in the volume to a probability. It should be noted that voxel-by-voxel addition was used to replace the most commonly used stacking by channel dimension as the hopping connection between the encoder and the decoder. As a result, parameters and GPU memory consumption were reduced without reducing model accuracy. GSPNet was an effective light-weight network that could be applied in clinical medical practice. It required only 0.24 M parameters and 23G FLOPs. Hence, GSPNet was the most light-weight model for medical image segmentation.

### Article retrieval

Articles published from January 2000 until now were retrieved from Pubmed, Web of Science core library, Nature, and Science Direct databases. The keywords included early ERCP, delayed ERCP, ABP, laparoscopy, and cholecystectomy, all which were connected by “or” and “and”. The language of articles was not restricted during the retrieval.

### Inclusion and exclusion criteria

The inclusion criteria were as follows.

A. Domestic and foreign articles on the comparison of the safety and effectiveness of early ERCP and delayed ERCP.
B. Articles with evaluable safety and effectiveness of early ERCP and delayed ERCP in direct or indirect way.
C. Articles with at least 15 included samples.
D. Articles with analyzable data, such as relative risk (RR) or odds ratio (OR) and 95% confidence interval (CI).

The exclusion criteria were as follows.

A. Reviews, conference reports, empirical lectures, case reports, and comments.
B. Articles unrelated to the research topic.
C. Articles without control group or comparability between samples in different groups.
D. The articles with the highest quality among the articles published with the same data.
E. Articles with small sample size.
F. Articles with unclear research type and incorrect randomized controls.
G. Articles with duplicate reports.
H. Incomplete articles with inaccessible full text after the contact with the author.
I. Articles from which no effective outcome data could be extracted.
J. Articles that met profession-related exclusion criteria.

### Evaluation of article quality

2 researchers independently read the retrieved articles and they were required to read the full text and extract relevant data from them. Any disagreement or dispute between them needed to be resolved through discussion or the assistance of a third researcher. Jadad was utilized to evaluate the quality of the included articles from the following perspectives.

A. Whether the article was RCT.
B. Whether appropriate random method was adopted.
C. Whether double blind experiment was carried out.
D. Whether appropriate double blind experiment was implemented.
E. Whether lost to follow-up or withdrawal occurred during the research and the reasons were explained. Whether intention-to-treat analysis was performed in the article.

“Yes” was scored 1 point and “No” was scored 0 point. The total score was 5 points. Low-quality articles were scored 2 points or less, while high-quality articles were scored more than 2 points.

After that, Cochrane evaluation handbook 4.2.6 was used to evaluate the quality of articles in the following aspects.

A. Whether the article was RCT.
B. Whether there was allocation concealment.
C. Whether blind experiment was carried out.
D. Whether outcome data were complete.
E. Whether there was selective reporting of results.
F. Whether there were other bias.

### Data extraction

Two researchers independently read articles and preliminarily determined whether articles were case controls or cohort studies and whether data were complete. According to the requirements of the meta-analysis, all related articles that met the inclusion criteria were screened and the quality of each of them was evaluated. The articles with duplicate reports and poor quality and unusable articles with little data were eliminated. Then, data were extracted according to the established table. In addition, database was set up and data were checked. If the reports in articles were incomplete, whether they were usable should be determined by contacting the author. Unusable articles were excluded from this research. If there was a disagreement between the two researchers, it should be settled through the discussion with a third party. After the full text was obtained, data were extracted and input into Microsoft Excel for sorting. The extracted data indicators included the basic information about the included articles (titles, research types, the first author, and publication year), the basic data on research objects (grouping methods, sample size, patient age, and others), and outcome indicators (uric acid, hemoglobin, bilirubin, and neonatal low body weight).

### Data extraction

The research results of all articles were clearly displayed in forest plots and the articles in the corresponding CIs were combined. No overlap between CIs of the results of all articles suggested statistical heterogeneity between articles. With acceptable heterogeneity, random models were combined with fixed models for further subgroup analysis. According to different designs, the models were divided into different subgroups. Next, different natures of the sizes of all subgroups were investigated. To deal with heterogeneity rather than different natures, they could be ignored when the heterogeneity between articles was non-negligible. The combined statistical model of the statistical model was selected.

Sensitivity analysis was implemented through the investigation into whether single article affected the general result of the combination. The articles included in this research were removed one by one and the remaining results were combined to compare the combined results with respective results to determine if they were the same. In general, integrated research was affected in the following two cases.

A. If an article was deleted, the estimate value of the effect size of integrated combination was another value other than 95% of composite effect size. If an article was deleted, there were apparently different results.
B. Insignificant difference in the influence of an article on overall outcome revealed the sensitivity of combined results and unstable outcome. Otherwise, the combined results were sensitive and stable. The conclusion was correct.

### Statistical analysis

Review Manager5.3 was utilized for the meta-analysis of the extracted data on included articles. In addition, I2 and P values in Peto test were used to analyze the heterogeneity of the extracted indicators. I2 ≥ 50% or *P* ≤ 0.05 suggested notable heterogeneity. In this case, random effect model was used. I2 < 50% or *P* > 0.05 revealed no apparent heterogeneity. Hence, fixed effect model was employed. What’s more, subgroup analysis was performed to analyze the sensitivity of the extracted indicators of included articles. Binary variable was described with RR, OR, or risk difference (RD). Continuous variable was expressed as weighted mean difference (WMD) or standard mean difference (SMD). All effect sizes were denoted by 95%CI. *P* < 0.05 demonstrated that the differences between groups suggested statistical significance.

## Results

### Features of ABP images

**Fig. 2:**
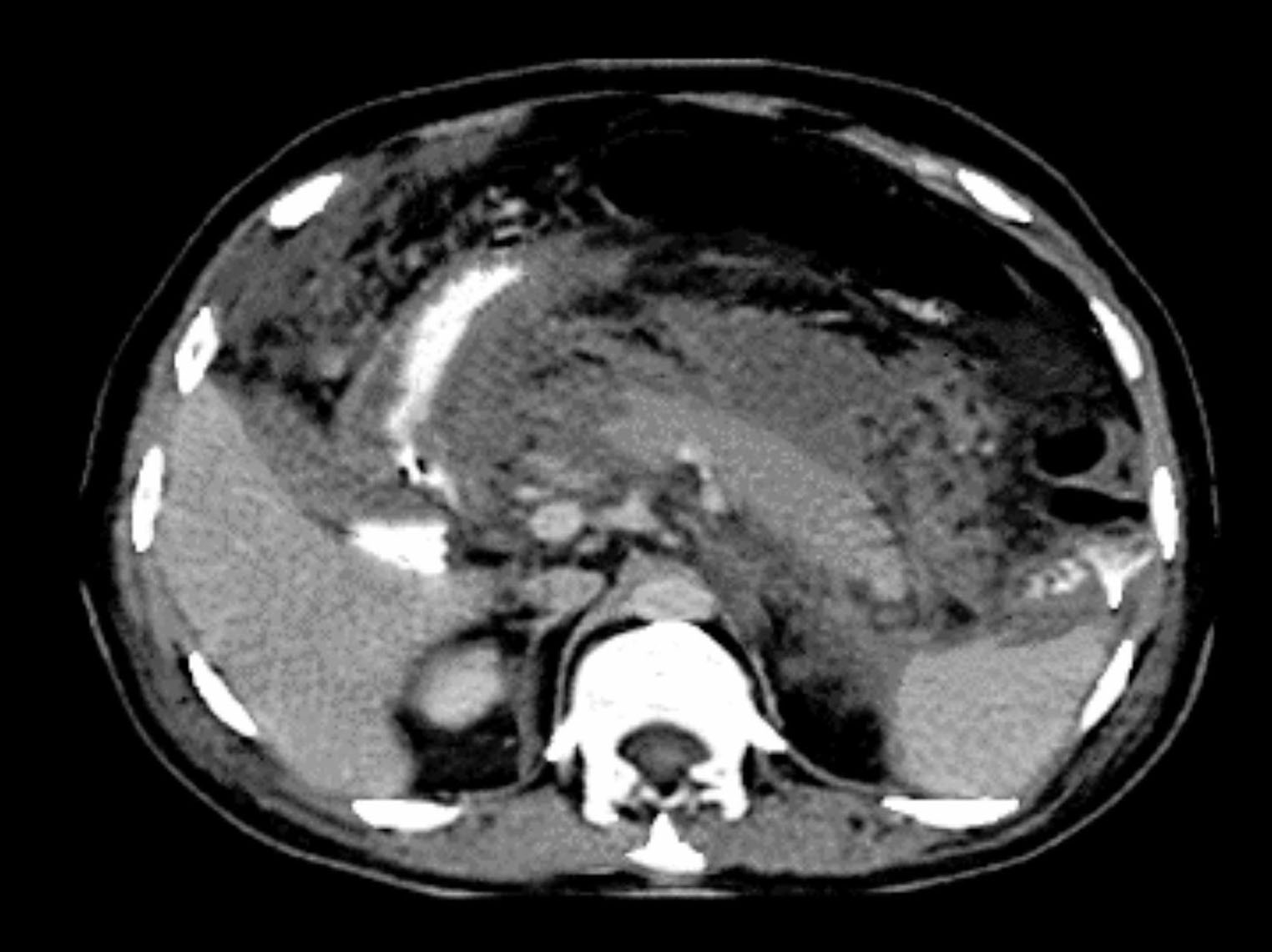
CT image of AP

AP is divided into interstitial edema pancreatitis and necrotizing pancreatitis. Patients with necrotizing pancreatitis suffer from pancreas parenchyma and peripancreatic tissue necrosis. Computed tomography (CT) was adopted to evaluate imaging changes. It was demonstrated that enhanced signals appeared in pancreas and pancreas was normal. However, peripancreatic necrosis occurred, including different amounts of liquid and non-liquid components (Fig. 2).

### Article retrieval results and general data analysis

**Fig. 3:**
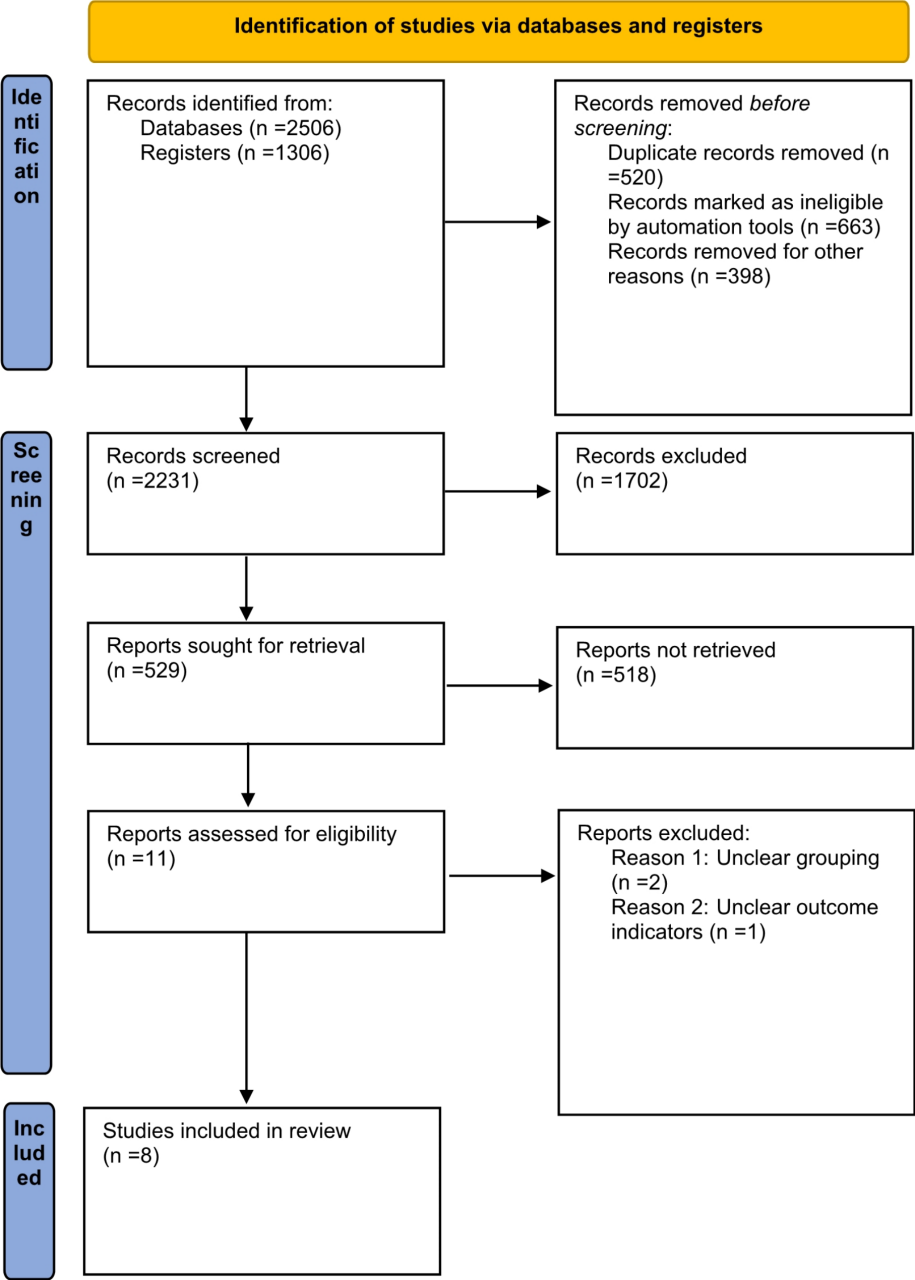
Document retrieval process

A total of 3,812 related articles were retrieved. Among all retrieved articles, 2,506 of them were retrieved from Pubmed, Medline, EMbase, EBSCO, and other databases, 1,306 were retrieved from Registers, and 1,532 duplicate articles were excluded. Then, 1,603 articles that didn’t met the inclusion criteria were eliminated by reading titles and abstracts. In addition, 675 articles were removed by reading the full texts. Finally, 8 eligible articles were included. Specific article screening procedure was presented in Fig. 3 and the details of articles were displayed in Table 1.

**Table 1:** Basic data on the included articles

The first author	Number of cases	Controls	Publication year	Countries	Regions
Oría A [9]	51	51	2007	Argentina	South America
Chen P [10]	21	32	2010	China	Asia
da Costa DW [11]	128	136	2015	Netherlands	Europe
Mueck KM [12]	49	48	2019	U.S.	North America
Riquelme F [13]	26	26	2019	U.S.	North America
Jee SL [14]	38	34	2016	Malaysia	Asia
Davoodabadi A [15]	104	104	2020	Iran	Asia
Aboulian A [16]	25	25	2010	U.S.	North America

### Evaluation of bias risk of included articles

**Fig. 4:**
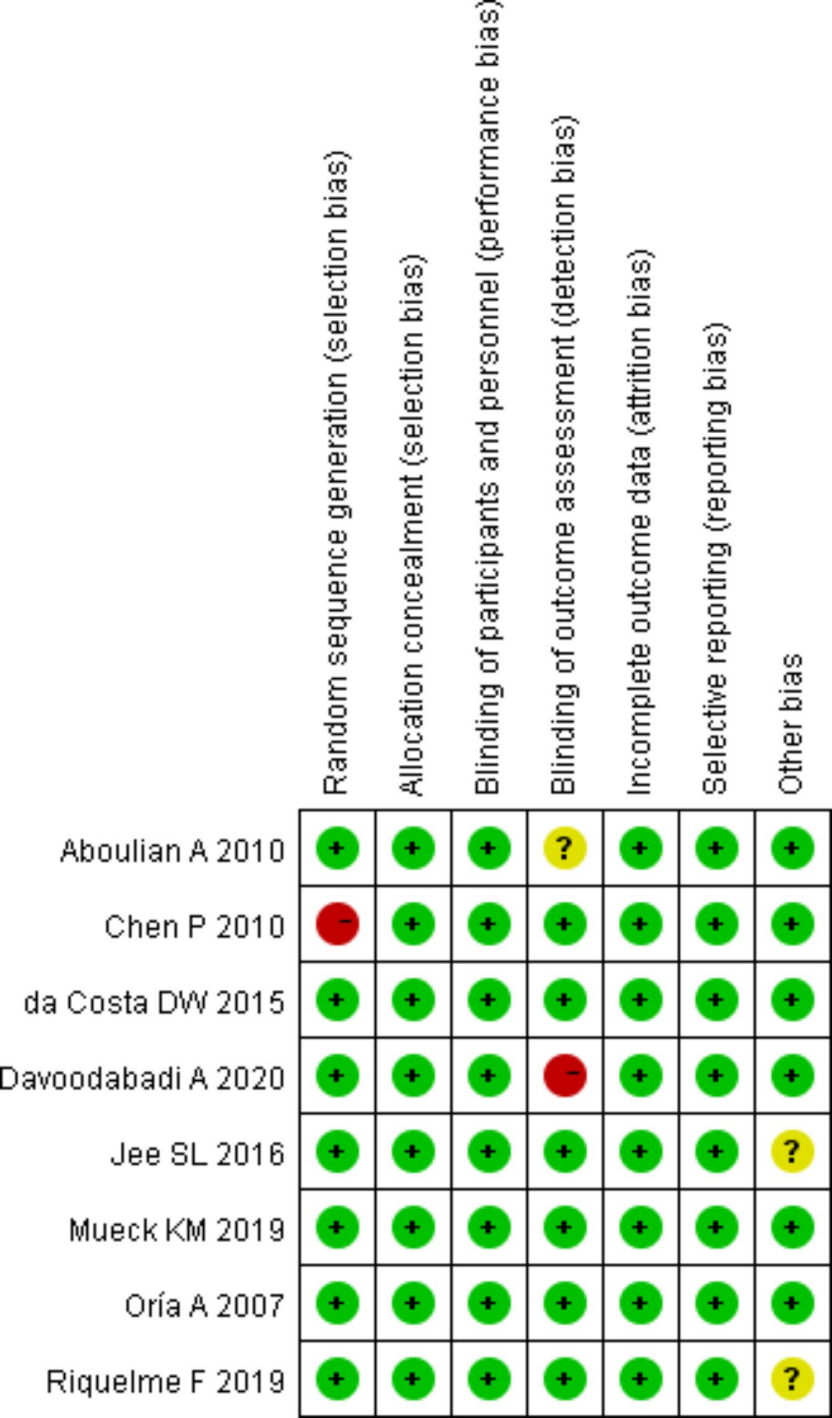
Evaluation of bias risk of included articles

**Fig. 5:**
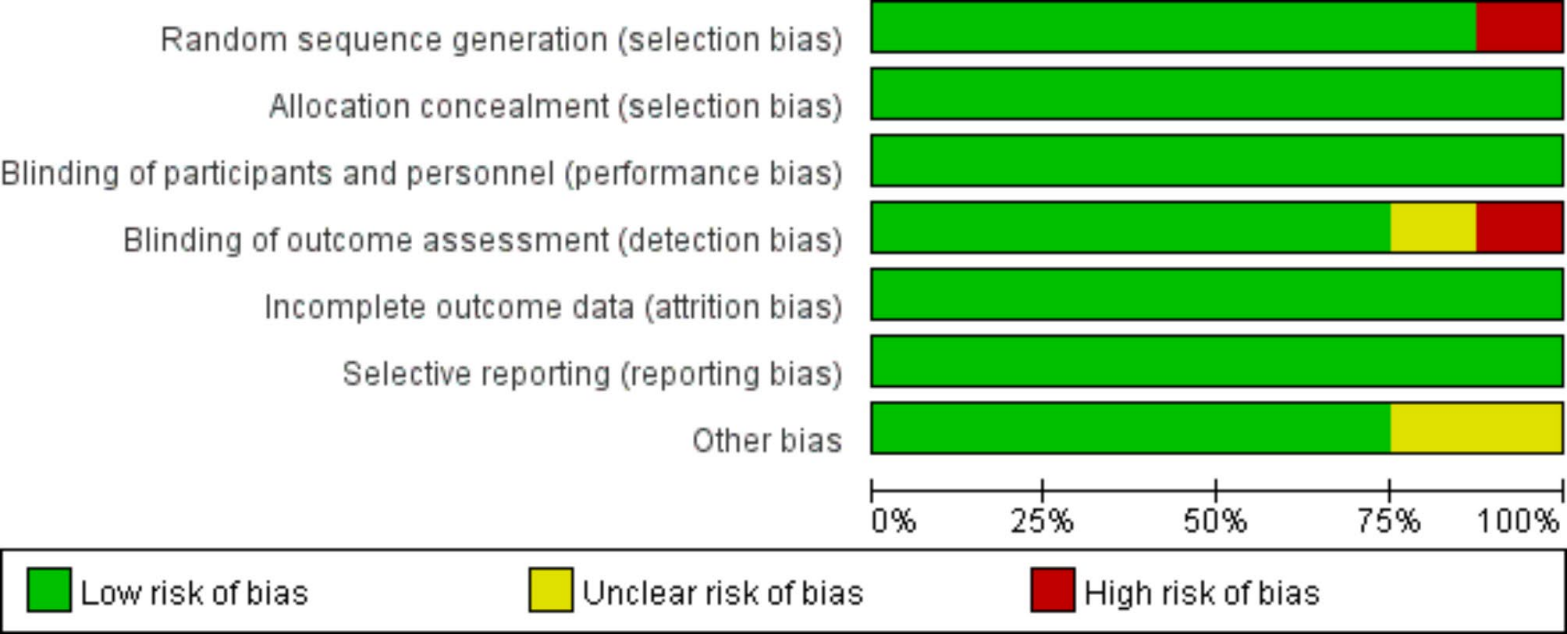
Bar charts of the evaluation of bias risk of included articles

Cochrane Handbook 5.3 system evaluation handbook was used to evaluate the bias risk of 7 included articles. After that, bias risk diagrams were output (Figs. 4 and 5) and expressed with Review Manager 5.3.

### Analysis of death among patients in two groups

**Fig. 6:**
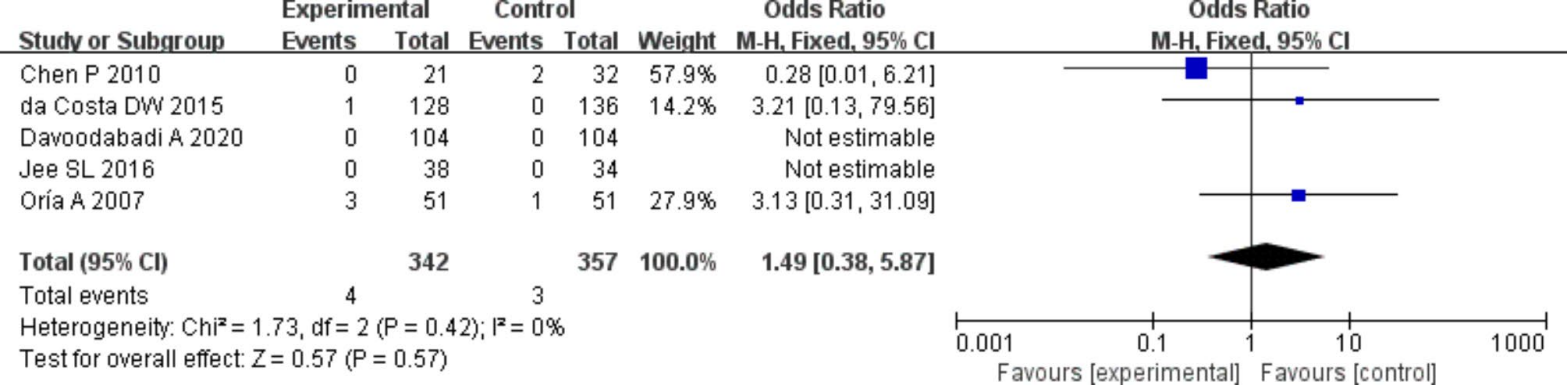
Forest plot of the comparison of death in two groups. *Note* SE and OR represented standard error and odds ratio, respectively

**Fig. 7:**
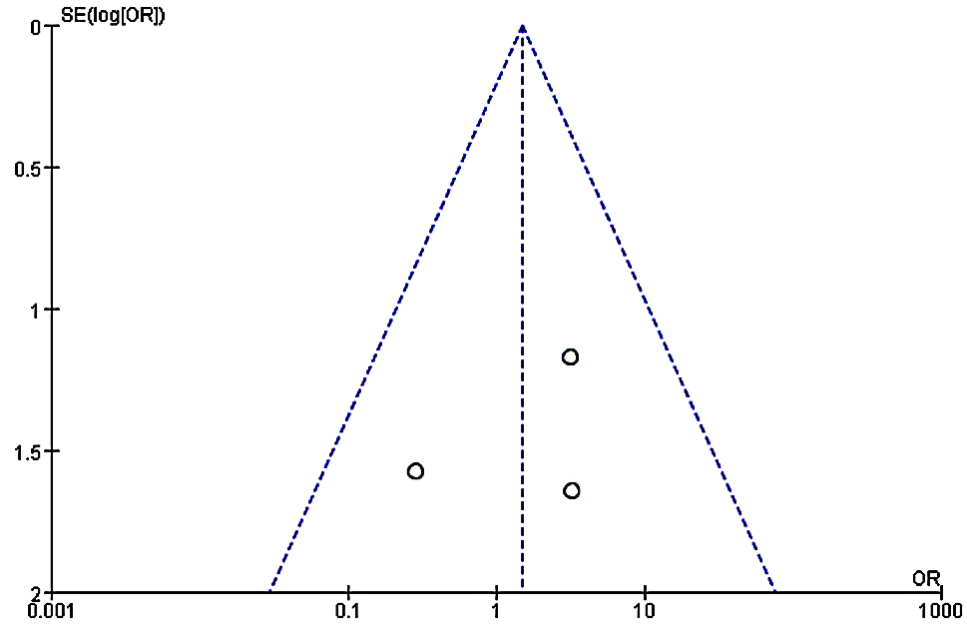
Funnel plot of the comparison of death in two groups

The death among patients in the two groups were compared and analyzed based on the investigation into 5 included articles. According to the meta-analysis of the death in the two groups, 95%CI amounted to 1.49[0.38,5.87], Z = 0.57, and I2 = 0%. According to I2 = 0% (< 50%), fixed effect model was employed and no heterogeneity was detected (*P* = 0.0.42, I2 < 50%). It was revealed that no statistical significance was detected in the difference in the number of dead patients between experimental and control groups. Figure 6 was the forest plot of the comparison and analysis of the death in the two groups. As illustrated in Fig. 7, the funnel plot was generally symmetric and most data were on either side of the central axis, which suggested that publication bias was effective.

### Incidence of postoperative complications

**Fig. 8:**
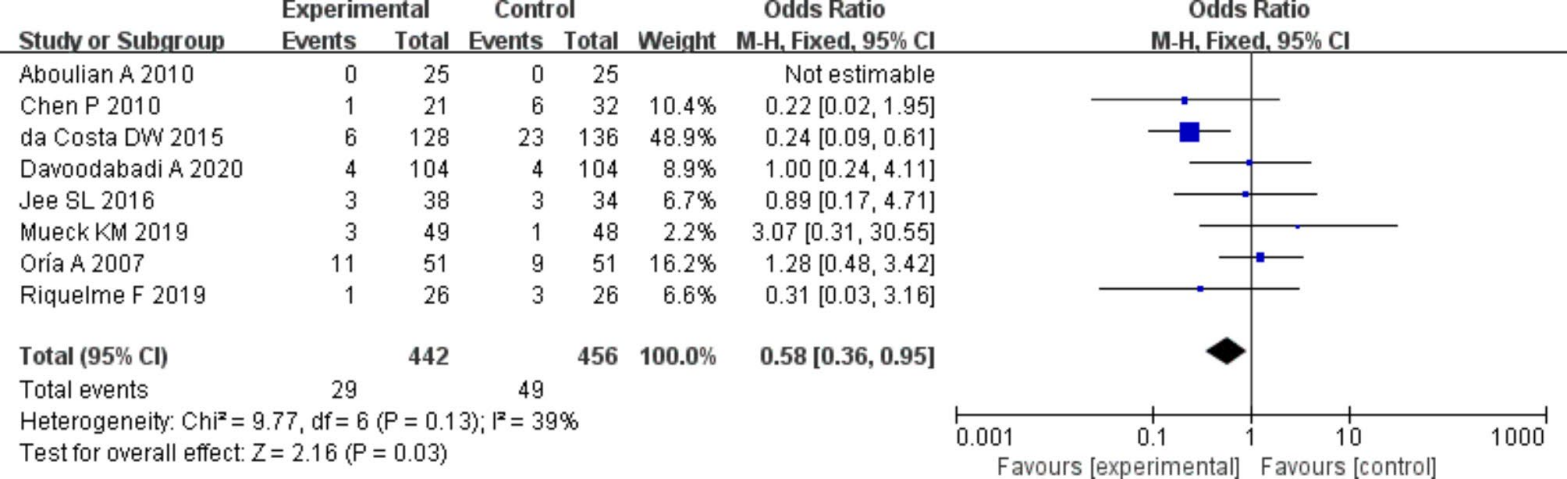
Forest plot of the comparison of the incidence of postoperative complications in two groups. *Note* SE and OR represented standard error and odds ratio, respectively

**Fig. 9:**
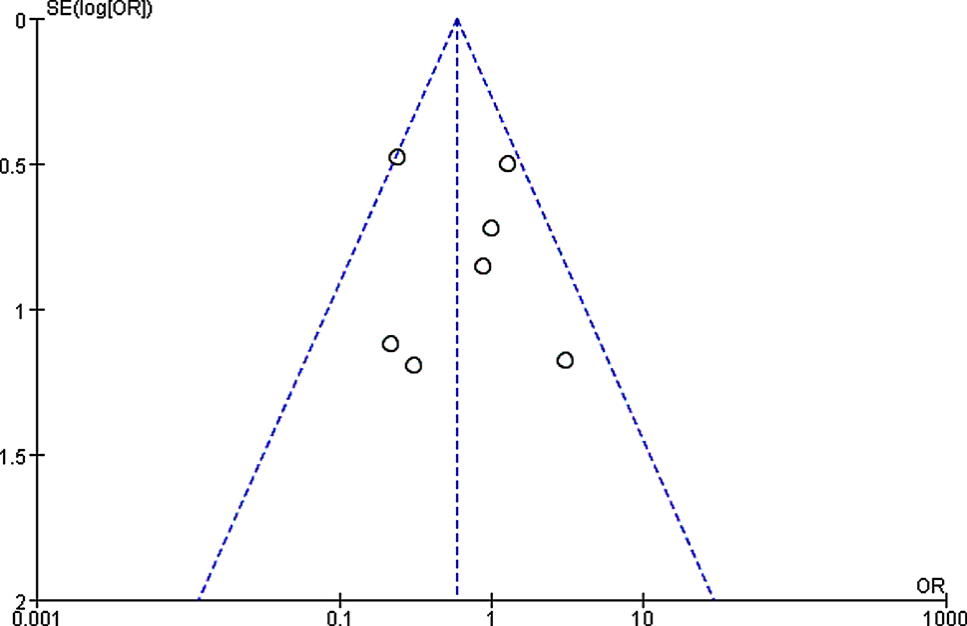
Funnel plot of the comparison of the incidence of postoperative complications in two groups

The incidence of complications among patients in the two groups were compared and analyzed based on the investigation into 8 included articles. According to the meta-analysis of the incidence of complications in the two groups, 95%CI amounted to 0.58[0.36,0.95], Z = 2.16, and I2 = 39%. According to I2 = 39% (< 50%), fixed effect model was employed and no heterogeneity was detected (*P* = 0.1 > 0.05, I2 < 50%). It was revealed that no remarkable difference was no detected in the incidence of complications between experimental and control groups. Figure 8 was the forest plot of the comparison and analysis of the incidence of complications in the two groups. As illustrated in Fig. 9, the funnel plot was generally symmetric and most data were on either side of the central axis, which suggested that publication bias was effective.

### Hospitalization duration

**Fig. 10:**
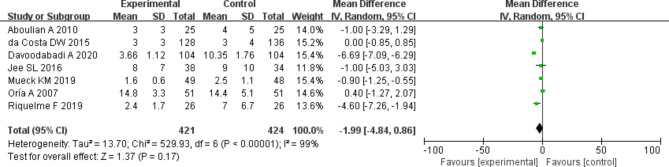
Forest plot of the analysis of the hospitalization duration in two groups. *Note* SE and OR represented standard error and odds ratio, respectively

**Fig. 11:**
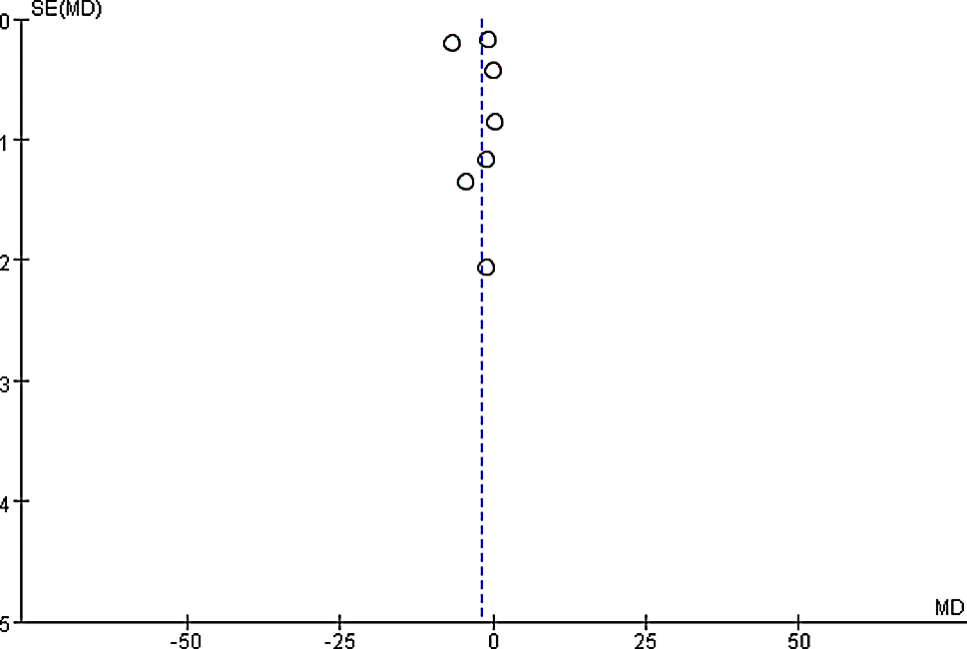
Funnel plot of the analysis of the hospitalization duration in two groups. *Note* SE and OR represented standard error and odds ratio, respectively

The hospitalization duration among patients in the two groups were compared and analyzed based on the investigation into 7 included articles. According to the meta-analysis of the hospitalization duration in the two groups, 95%CI amounted to -1.99[-4.84,0.86], Z = 1.37, and I2 = 99%. According to I2 = 99% (> 50%), random effect model was employed and heterogeneity was detected (*P* < 0.000001, I2 > 50%). It was revealed that remarkable difference was detected in the hospitalization duration between experimental and control groups. Figure 10 was the forest plot of the comparison and analysis of the hospitalization duration in the two groups. As illustrated in Fig. 11, the funnel plot was generally symmetric and most data were on either side of the central axis, which suggested that publication bias was effective.

### Operation time

**Fig. 12:**
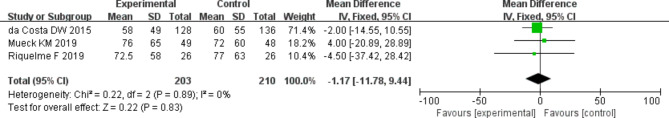
Forest plot of the comparison and analysis of the operation time in two groups

**Fig. 13:**
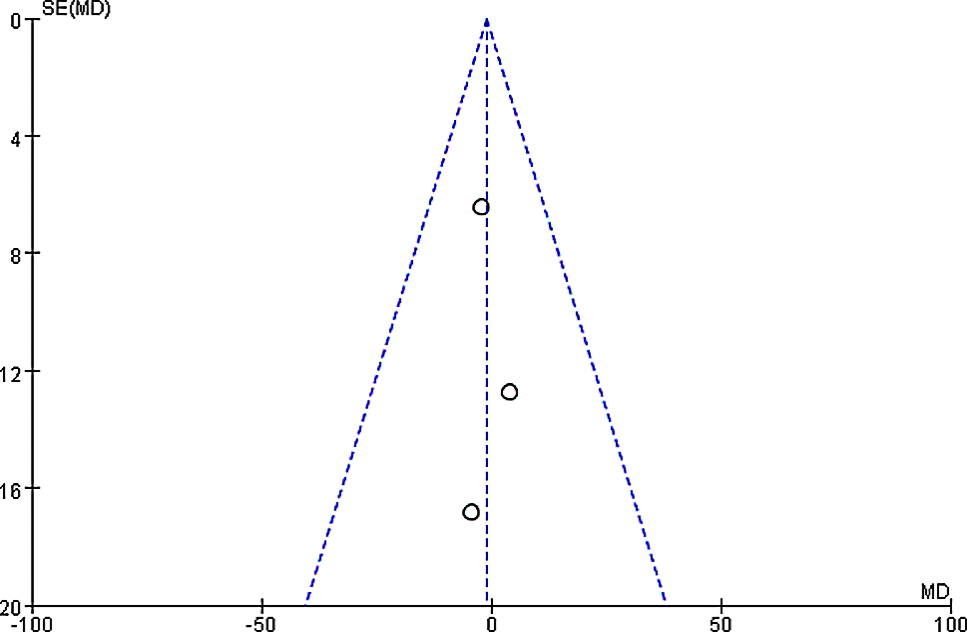
Funnel plot of the comparison and analysis of the operation time in two groups. *Note* SE and OR represented standard error and odds ratio, respectively

The operation time among patients in the two groups were compared and analyzed based on the investigation into 3 included articles. According to the meta-analysis of the operation time in the two groups, 95%CI amounted to -1.17[-11.78,9.44], Z = 0.22, and I2 = 0%. According to I2 = 0% (< 50%), fixed effect model was employed and no heterogeneity was detected (*P* = 0.89, I2 < 50%). It was revealed that no remarkable difference was detected in the operation time between experimental and control groups. Figure 12 was the forest plot of the comparison and analysis of the operation time in the two groups. As illustrated in Fig. 13, the funnel plot was generally symmetric and most data were on either side of the central axis, which suggested that publication bias was effective.

## Discussion

AP is one of the commonest acute abdomens in surgical clinical practice. At present, the identification of the cause of AP is the key determinant of clinical treatment of AP. In China, pancreatitis is mainly divided into biliary pancreatitis and alcoholic pancreatitis. ABP accounts for as high as 80% in AP. The main causes of ABP include cholecystolithiasis, bile duct stones, acute cholangitis, sclerosing cholangitis, ascariasis of biliary tract, and choledochal cyst. The commonest causes include cholecystolithiasis and bile duct stones [17]. No consensus has been reached on the pathogenesis of ABP. At present, the mainstream view is that biliary pancreatitis is caused by cholecystolithiasis, intrahepatic and extrahepatic bile duct stones, or biliary sludge. In most cases, biliary stones and sludges enter common bile duct and then bile flows back into pancreatic duct trough the common pathway of common bile duct and the main pancreatic duct. After that, bile salt enters pancreatic duct, which leads to acinar cell necrosis or high pressure and rupture of pancreatic duct. As a result, pancreatic juice enters the tissue around acini and then pancreatitis occurs. In addition, it is believed that gallbladder or gall stone enters or indirectly enters duodenum, which results in sphincter edema. Spasm-induced functional obstruction is also a main cause of ABP. In general, the pathogenesis of biliary pancreatitis was still unclear, while it is very certain that pathogenesis is harmful for health among patients [18]. According to related clinical statistical results, the mortality among patients with severe AP reached 40% though the overall fatality is lower than 5%. Besides, 11–32% patients suffered from recurrent pancreatitis after they were diagnosed with this disease for the first time. Recurrent pancreatitis with unknown cause significantly affects quality of life among patients and causes heavy economic burdens to society and families [19].

At present, early ERCP is not recommended for the treatment of ABP and patients without intestinal obstruction and cholangitis in American and Chinese guidelines. In daily clinical practice, when the existence of stones is uncertain, EUS is usually used to ignore unnecessary ERCP and lead to a good clinical course. Liu et al.’s research shows that EUS can safely replace diagnostic ERCP in the treatment of patients with common bile duct stones, with higher success rate and higher sensitivity in detecting gallstones, and the incidence of both is equivalent. In these cases, early ERCP is unnecessary [20, 21]. AP patients with choledocholith incarceration are advised to receive ERCP within 24 h after admission. Coutinho et al. obtained the research results that early ERCP can reduce local adverse events, shorten the time of pain relief, and reduce axillary temperature, hospitalization time and expenses of patients with acute biliary pancreatitis. It shows that early ERCP of ABP is desirable [22]. According to the treatment guideline, patients with severe pancreatitis are advised to undergo ERCP after relevant indicators recover (6 weeks after the treatment) [23]. However, the therapeutic effect of early ERCP on biliary pancreatitis is very controversial in clinical practice. The traditional view is that the treatment is safer and more effective after clinical symptoms and experimental indicators are improved. In this case, the incidence of complications and mortality among patients is effectively reduced. What’s more, it is believed that adhesion and anatomical disorder caused by inflammation and edema may apparently increase the difficulty of dissection and the incidence of related complications in early stage. Some research show that delayed ERCP increases the incidence of recurrent biliary events versus early ERCP, such as recurrent pancreatitis, cholecystitis, and biliary colic. The results of Navaneethan et al. showed that 37.8% of patients had recurrent AP, and the potential causes of recurrent cholangitis were stent occlusion and incomplete removal of biliary stones during exponential ERCP. The study also pointed out that the serum bilirubin level of patients with delayed ERCP is high. Although it is uncertain, these patients may get worse with time [24]. More and more relevant research demonstrates that early ERCP can not only effectively shorten the hospitalization duration, but also doesn’t increase the incidence of relevant complications during perioperative period versus delayed ERCP [14, 22]. In addition, early ERCP reduces the incidence of relevant biliary events. The research result obtained by Halász et al. is that if ERCP is carried out later, the incidence of local complications will increase, which is less than 24 h: 21.1%, 24–48 h: 23.4% and more than 48 h: 37.2% [25].

In general, no clinical consensus has been reached on the therapeutic effect and safety of early ERCP in biliary pancreatitis [26]. ERCP has high values in the diagnosis and treatment of pancreatitis. It is not only a treatment method but also a diagnosis approach. In recent years, it has been widely applied and developed in medical field with the continuous and rapid development of computer technology. Light-weight deep learning model is one of the recent noteworthy computer network technologies. According to the pathological images to be diagnosed, the model selected the samples similar to the image features from numerous databases. The selected samples came with their own labels to help doctors diagnose diseases. As a result, diagnostic efficiency and accuracy were dramatically improved [27]. At present, the technology has been applied in related fields, such as the segmentation of tumor image lesions. In contrast, it has been rarely applied in the diagnosis and treatment of pancreatitis [28, 29]. Therefore, light-weight deep learning model was innovatively combined with ERCP and adopted for the diagnosis and treatment of pancreatitis. What’s more, the articles on the treatment of biliary pancreatitis with early ERCP were retrieved and collected. A meta-analysis of the safety of the above therapy was performed in terms of fatality, the incidence of complications, hospitalization duration, and operation time. The research findings revealed that no remarkable differences were detected in mortality, the incidence of complications, and operation time between experimental and control groups, while hospitalization duration in experimental group was notably shorter than that in control group. The results showed that there was no significant difference in the safety of early ERCP treatment for biliary pancreatitis and late ERCP treatment for biliary pancreatitis, but early ERCP treatment could significantly shorten the hospital stay.

## Conclusions

This research was conducted to investigate the safety of early ERCP in the treatment of ABP. The articles on the treatment of ABP with early ERCP were retrieved and collected and a meta-analysis of the safety of the above therapy was performed in terms of fatality, the incidence of complications, hospitalization duration, and operation time. According to the research findings, no remarkable differences were detected in mortality, the incidence of complications, and operation time between experimental and control groups, while hospitalization duration in experimental group was notably shorter than that in control group.

It was concluded that early ERCP treatment is as safe as late ERCP treatment for biliary pancreatitis, and could apparently shorten hospitalization duration. Hence, the therapy was worthy of clinical promotion. In addition, ERCP images were collected and processed with light-weight deep learning model. The research findings provided reference and basis for clinical treatment of relevant diseases. Nevertheless, there are some limitations and deficiencies in this research. For instance, the incidence of postoperative biliary events among patients in the two groups was not compared due to the limitation of objective conditions during the meta-analysis. Consequently, the conclusion was not comprehensive enough. In follow-up study and work, the above defects will be overcome and the research will be further improved.

### Electronic supplementary material

Below is the link to the electronic supplementary material.

Supplementary Material 1

## Data Availability

The figures and tables used to support the findings of this study are included in the article.

## Abbreviations

ABP: Acute biliary pancreatitis
ERCP: Endoscopic retrograde cholangiopancreatography
AP: Acute pancreatitis
ASP: Acute severe pancreatitis
RCTs: Randomized controlled trials
EST: Enriched supportive therapy
MABP: Mean arterial blood pressure
GSPNet: Ghost spatial pyramid net
GPU: Graphics processing unit
RR: Relative risk
OR: Odds ratio
CI: Confidence interval
RD: Risk difference
WMD: Weighted mean difference
SMD: Standard mean difference
CT: Computed tomography
